# Key roles of necroptotic factors in promoting tumor growth

**DOI:** 10.18632/oncotarget.7924

**Published:** 2016-03-05

**Authors:** Xinjian Liu, Min Zhou, Ling Mei, Jiaying Ruan, Qian Hu, Jing Peng, Hang Su, Hong Liao, Shanling Liu, WeiPing Liu, He Wang, Qian Huang, Fang Li, Chuan-Yuan Li

**Affiliations:** ^1^ Department of Dermatology, Duke University Medical Center, Durham, North Carolina 27710, USA; ^2^ Cancer Center, Shanghai General Hospital, School of Medicine, Shanghai Jiaotong University, Shanghai 201620, China; ^3^ State Key Laboratory of Oncogenes and Related Genes, Shanghai Cancer Institute, Renji Hospital, School of Medicine, Shanghai Jiao Tong University, Shanghai 200032, China; ^4^ Department of Obstetrics & Gynecology, West China Second University Hospital, Sichuan University, Chengdu, Sichuan 610041, China; ^5^ MOE Key Laboratory of Obstetric & Gynecologic and Pediatric Diseases and Birth Defects, West China Second University Hospital, Sichuan University, Chengdu, Sichuan 610041, China; ^6^ Laboratory of Cell and Gene Therapy, West China Institute of Women and Children's Health, West China Second University Hospital, Sichuan University, Chengdu, Sichuan 610041, China; ^7^ Laboratory of Genetics, West China Institute of Women and Children's Health, West China Second University Hospital, Sichuan University, Chengdu, Sichuan 610041, China; ^8^ Department of Pathology, West China Hospital, Sichuan University, Chengdu, Sichuan 610041, China; ^9^ Department of Pharmacology and Cancer Biology, Duke University Medical Center, Durham, North Carolina 27710, USA

**Keywords:** necroptosis, RipK1, RipK3, MLKL, tumor growth

## Abstract

Necroptotic factors are generally assumed to play a positive role in tumor therapy by eliminating damaged tumor cells. Here we show that, contrary to expectation, necroptotic factors RIPK1, RIPK3, and MLKL promote tumor growth. We demonstrate that genetic knockout of necroptotic genes *RIPK1, RIPK3, or MLKL* in cancer cells significantly attenuated their abilities to grow in an anchorage-independent manner. In addition, they exhibited significantly enhanced radiosensitivity. The knockout cells also showed greatly reduced ability to form tumors in mice. Moreover, necrosulfonamide (NSA), a previously identified chemical inhibitor of necroptosis, could significantly delay tumor growth in a xenograft model. Mechanistically, we show that necroptoic factors play a significant role in maintaining the activity of NF-κB. Finally, we found that high levels of phosphorylated MLKL in human esophageal and colon cancers are associated with poor overall survival. Taken together, we conclude that pro-necroptic factors such as RIPK1, RIPK3, and MLKL may play a role in supporting tumor growth, and MLKL may be a promising target for cancer treatment.

## INTRODUCTION

Cell death mechanisms are of central importance in cancer biology because cancer development depends on evasion of cell death while treatment of cancer depends on effective elimination of malignant cells. Traditionally necrosis is generally thought of as an unregulated form of cell death that is triggered by external agents such as exposure to viruses, physical trauma, or cytotoxic agents such as radiation. However, recently it was recognized that necrosis, which is characterized by the presence of an intact nucleus and swelling and puncturing of the cytoplasmic membrane, can also occur in a highly regulated manner. Such regulated necrosis is termed programmed necrosis [1, 2] or necroptosis [3–5].

In necroptosis, a well-coordinated cascade of molecular events leading to eventual puncturing of cytoplasmic membrane and cell death are involved [5–8]. Among the molecular factors involved in necroptosis, RIPK1 (receptor-interacting serine/threonine-protein kinase 1) [7], RIPK3(receptor-interacting serine/threonine-protein kinase 3) [9–11], and MLKL (mixed lineage domain-like) [8, 12–14] are the most well-characterized. They are involved in the TNF-α induced necroptosis, which is the experimental model most investigators use when studying programmed necrosis. Previous studies have indicated that upon activation (by ligands such as TNF-α) the death receptor forms an intracellular complex (complex I) that consists of multiple proteins such as TRADD, RIPK1, TRAF2, TRAF5, cIAP1, and cIAP2 [15–18]. Complex I plays a key role in the activation of the transcription factor NF-κB that promotes cellular survival [19, 20]. TNFR1 internalization after TNF-α binding leads to the formation of complex II, which contains RIPK1, caspase 8, TRADD and FADD, that is responsible for caspase 8 activation and TNF-α induced apoptosis [20]. In TNF-α induced necrotic cell death, a similar complex is formed, which is called complex IIb [21] or the necrosome [22]. Activation of the necrosome is prevented by caspase 8, which can inactivate RIPK1 and RIPK3 by cleavage of the two kinases. On the other hand, deletion or inhibition of caspase 8 promotes necrosis [23–28]. When caspases are inactive or inhibited, RIPK1 can autophosphorylate and trans-phosphorylate RIPK3, which in turn phosphorylate MLKL. The three factors then form the so-called necrosome, which goes on to engage PGAM5 and DRP1 [29, 30] and migrate to the cellular membrane to cause its rupture and leakage, which leads to cell death.

Because necrosis has been traditionally linked to inflammation, it has been suggested that inhibition of necroptotic factors could be beneficial in treating disease involving pathological inflammation such as sepsis [31]. Published results appeared to be consistent with this suggestion [16, 32–34]. Furthermore, similar to apoptosis and autophagy, necroptosis has also been implicated in cancer treatment [35, 36]. For example, there are reports of involvement of necroptosis in cancer cells exposed to radiotherapy [37] and chemotherapeutic agents [38, 39]. In particular, some reports suggest that necroptosis activation should be a good approach to bypass anti-apoptotic mechanisms that are activated in many cancer cells and therefore should be positive for cancer control. However, currently there is still no consensus on the overall effect of necroptosis in tumor growth or treatment.

In the present study, we set out to define the role of necroptosis in tumor growth and treatment. By use of the CRISPR/Cas9 technology, we knocked out three genes shown to be essential for necroptosis: RIPK1, RIPK3, and MLKL. Our experiments in those cells revealed the surprising finding that necroptotic genes play key roles in sustaining anchorage-independent tumor growth and mediating tumor cell resistance to radiation rather than promoting cell death. In addition, we show that a small molecule inhibitor of MLKL could potently inhibit tumor growth in mice. We also show the tumor-promoting properties of the necroptotic factors are based on NF-κB. Furthermore, we also provide evidence that our findings are relevant in human cancer patients.

## RESULTS

### Genetic ablation of the necroptotic genes and its effects on cellular growth, necroptosis, and radiation sensitivity

**Figure 1:**
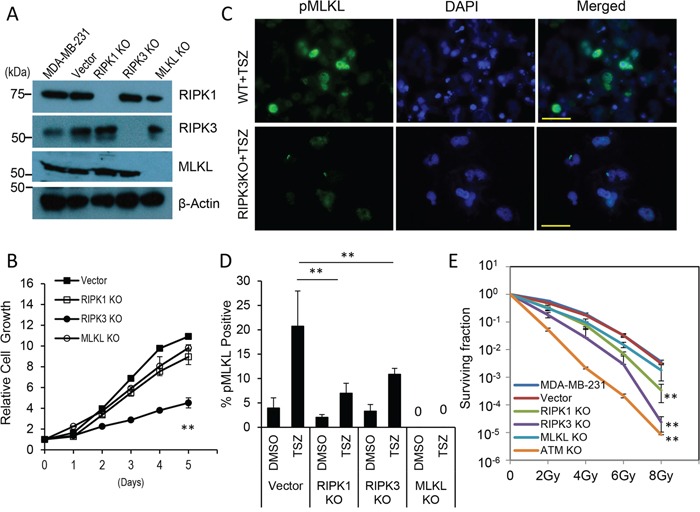
Effects of RIPK1-, RIPK3-, and MLKL- gene knockouts on MDA-MB-231 breast cancer cells *in vitro* **A.** Western blot analysis of clonal MDA-MB-231 cells with CRISPR/Cas9-mediated knockouts of *RIPK1*, *RIPK3*, and *MLKL* genes, respectively. **B.** Growth curve of MDA-MB-231-derived RIPK1*KO*, RIPK3*KO*, and MLKL*KO* cells and lentiCRISPR vector transduced of MDA-MB-231 cells as measured by use of the MTT assay. The error bars represent standard error of the mean (SEM, n=3, **, *p*<0.001, Student's t test). **C.** Immunofluorescence staining of phospho-MLKL (pMLKL) in TSZ (20 ng/ml TNFα, 100 nM Smac mimetic, 20 μM z-VAD-fmk) treated MDA-MB-231 vector control (top panels) and RIPK3 knockout (lower panels) cells. Scale bars represent 50 μm. **D.** Quantitative estimate of the percentage of pMLKL-positive cells in TSZ treated vector control-, RIPK1-, RIPK3- and MLKL- knockout MDA-MB-231cells. The error bars represent standard error of the mean (SEM, n=3, **, *p*<0.01, Student's t-test). **E.** Survival curve of MDA-MB-231 derived vector control, RIPK1*KO*, RIPK3*KO*, and MLKL*KO* cells after x-Ray irradiation. Cas9/CRISPR derived ATM-knockout cells (ATM *KO*) were used as controls. Error bars represent standard error of the mean (SEM, n=3, **, *p*<0.001, Student's t-test).

In order to evaluate the functions of the necroptotic factors, we used lentiviral vectors [40, 41], to deliver sgRNAs and Cas9 gene to knockout each of the three factors known to play a key role in necroptosis: RIPK1, RIPK3, and MLKL. A freely available online software [41] was used to design the sgRNA minigene sequences (see Supplementary Figure S1 and Table S1 for sgRNA sequences). The oligos were then synthesized and introduced into lentiCRISPR vectors [40]. Our results show that the efficiencies of knocking out the genes were quite high but varied according to different sgRNAs. After initial selection, we selected individual clones that had stably integrated the lentiCRISPR vector and expanded the clones for western blot analysis. Figure 1A shows results from representative clonal cell populations with successful knockout for each of the three necroptotic genes. Those clones that showed clear absence of target protein expression were further evaluated for target gene sequence mutations. Those clones that show unequivocal gene knockout were selected for further experiments. Supplementary Figure S1 shows sequencing results for representative MDA-MB-231 clones.

When cellular growth rates for the knockout cells were evaluated, we found that MDA-MB-231 cells with *RIPK1* and *MLKL* knockout showed minimal disruption in cellular growth while *RIPK3* knockout cells showed significant (*p*<0.001) slowdown when compared with vector-transduced cells despite still proliferating at a robust rate (Figure 1B). The cells were then evaluated for necroptosis by use of staining for phosphorylated MLKL, which has been identified as a marker for necroptosis [12]. Our results indicated that parental MDA-MB-231 cells went through a significant amount of necroptosis when treated with TNF-α in combination with a Smac mimetic and the pan-caspase inhibitor z-VAD-FMK (abbreviated as TSZ treatment, Figure 1C&1D). However, the amount of necroptosis was significantly reduced in cells with RIPK1 and RIPK3 knockout, as expected.

We further carried out clonogenic survival assays [42] to examine the radiation sensitivity of the cells with gene knockout. All three gene knockout cells showed decreased radiation resistance with RIPK3<RIPK1<MLKL (Figure 1E). In particular, RIPK3 knockout showed significantly increased radiation sensitivity. As a comparison, clonogenic survival data for MDA-MB231 cells with ATM gene knockout was shown (see Supplementary Figure S2 for data showing ATM gene knockout in MDA-MB-231 cells). ATM-deficient cells are some of the most radiation sensitive cells ever observed [43, 44]. Consistently, MDA-MB-231 ATMKO cells were almost 1000 fold more sensitive than parental MDA-MB-231 cells when exposed to 8 Gy of x-rays. Another interesting observation is that in addition to decreased colony numbers, *RIPK3* knockout cells also exhibited significantly diminished colony size (Supplementary Figure S3). While we do not understand the underlying mechanism for the small colony sizes in all three knockouts, we speculate that they might be caused by reduced growth factor secretion which manifests more prominently when the cells are sparsely populated and less when the cells were more densely seeded when their growth rates were measured (Figure 1B).

We further investigated apoptotic and necroptotic cell death pathways in the necroptotic gene knockout cells after irradiation. Our results show that radiation enhanced phosphorylation of MLKL in control MDA-MB-231 cells, indicating increased necroptosis (Figure S4A). However, radiation-induced MLKL phosphorylation was diminished in *RIPK1*-, *RIPK3*- knockout cells. When cleaved caspase 3, an established marker for apoptosis, was measured, irradiated *RIPK3*- and *MLKL*- KO cells appeared to have only slightly increased caspase3 activation when compared with vector transfected MDA-MB-231 cells (Supplementary Figure S4B), indicating increased apoptosis is not obvious in these cells. On the other hand, *RIPK1* knockout cells have clearly reduced caspase 3 activation.

### The effects of necroptotic gene knockout on anchorage-independent tumor cell growth *in vitro* and tumor formation *in vivo*

**Figure 2:**
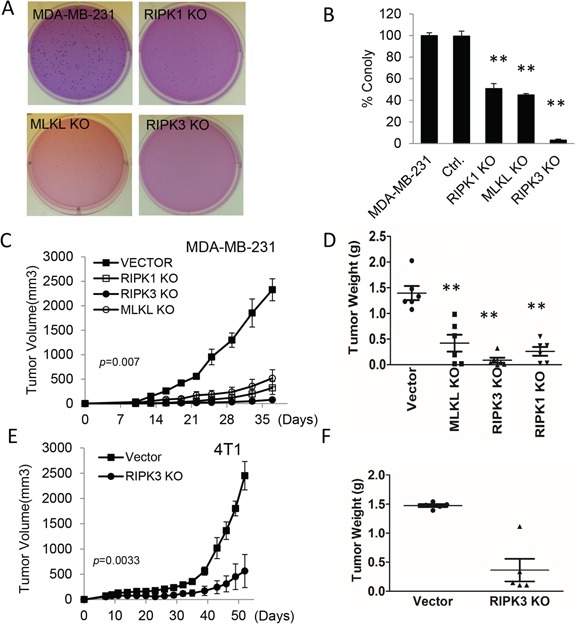
Effect of necroptotic gene deficiencies on the tumorigenicity of human and murine breast cancer cells **A.** Representative soft agar colony images of MDA-MB-231 derived vector control, RIPK1-, RIPK3-, and MLKL- KO cells. About 250 cells were plated into each well of 6-well plates. **B.** Quantitative estimate of the number of soft agar colonies of RIPK1-, RIPK3-, and MLKL- KO in MDA-MB-231 cells. (Error bars represent SEM, n=3, **, p<0.001, Student's t-test). **C.** Xenograft tumor growth in nude mice from MDA-MB-231 cells transduced with control vectors and those with knockouts in RIPK1, RIPK3, and MLKL. Error bars represent SEM, n=6, p=0.007, ANOVA. **D.** Tumor weigh distribution among different tumor groups upon termination of tumor growth experiments at day 37 post tumor cell injection. (**, p<0.001, Student's t-test). **E.** Effect of RIPK3 gene deficiency on the growth of 4T1 breast cancer cells in syngeneic Balb/C mice. Error bars represent SEM, n=5. *, p=0.0033, Student's t-test. **F.** Distribution of tumor weights upon termination of tumor growth on day 52. p<0.001, Student's t-test.

We next examined the effects of the necroptotic gene deficiencies on the tumorigenic abilities of the MDA-MB-231 cells. To measure the tumorigenic abilities of the cells *in vitro*, we used the well-established soft agar assay [45]. Our results indicate that vector-transduced MDA-MB-231 cells have very strong anchorage-independent growth ability (Figure 2A&2B). In contrast, *RIPK1-, RIPK3*-, and *MLKL*- knockout cells have significantly reduced soft agar clonogenic abilities with *RIPK3* knockout cells possessing the most decline (Figure 2A&2B). To confirm that our observation is not a cell line-specific phenomenon, we also carried out soft agar growth assay by use of the mouse breast cancer 4T1 cells with the *RipK3* gene knockout. Our results show that *RipK3* knockout in 4T1 cells also reduced the colony forming abilities of host cells in soft agar significantly (Supplementary Figure S5), consistent with results obtained with MDA-MB-231 cells.

We further carried out tumor growth experiments by use of vector-transfected MDA-MB-231 cells and necroptotic gene knockout cells in nude mice. Our results show that each of the three gene knockout cell lines showed significant growth delay in nude mice (Figure 2C) when compared with parental MDA-MB-231 cells transduced with vector control, consistent with observations made in soft agar assays. Measurement of tumor weights at the end of the experiments confirmed the growth delays (Figure 2D). Immunohistochemistry analysis of phosphorylated MLKL, which is an established marker for necroptosis [12], showed that in xenograft tumors established from control, RIPK1KO, and RIPK3KO cells, there was clear pMLKL staining (Supplementary Figure S6A), consistent with some necroptosis being present in the tumors. Phosphorylated MLKL staining was the strongest in control cells and significantly weaker in *RIPK1* and *RIPK3* knockout cells (Figure S6B), suggesting reduced necroptosis in those two types of tumors.

We next carried out tumor growth experiment by injecting vector-transduced or *RipK3*KO mouse breast cancer 4T1 cells into syngeneic Balb/C mice. Our results further confirmed the important role of RIPK3 in maintaining *in vivo* tumor growth in the 4T1 model with *RipK3* knockout 4T1 tumor cell growing at a significantly slower rate than vector control cells (Figure 2E). The growth delay data was also confirmed by tumor weight measurements (Figure 2F).

### Effects of a MLKL inhibitor on tumor cell growth in soft agar and in mice

**Figure 3:**
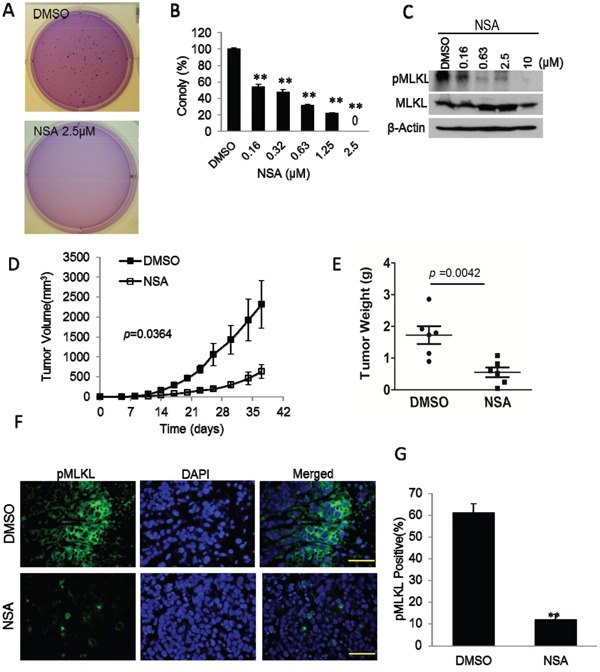
Inhibition tumor growth by MLKL inhibitor necrosulfonamide (NSA) **A.** Representative image of soft agar colony growth from MDA-MB-231 cells treated with vehicle (DMSO) or necrosulfonamide (NSA). About 250 cells were plated into each well of 6-well plates for the soft agar experiments. **B.** Quantitative estimate of soft agar colony forming abilities of vehicle(DMSO) and NSA(at different concentrations) treated MDA-MB-231 cells. Error bars represent SEM (n=3, **, p<0.001, Student's t-test). **C.** NSA-mediated suppression of phosphorylation of MLKL (pMLKL) in MDA-MB-231 cells. Cells were treated with NSA at different concentrations for 72 hrs and then lysed for western blot analysis. Top panel shows levels of phosphorylated MLKL while the middle panel shows total MLKL levels. **D.** Effect of NSA administration on MDA-MB-231 xengraft tumor growth in nude mice. NSA was administered by intraperitoneal injection (at 3.7 mg/kg/day) on a daily basis for 7 days. Error bars represent SEM (n=6, p=0.0364, ANOVA). **E.** Distribution of MDA-MB-231 xenograft tumor weights upon termination of tumor growth experiments at 37 days post tumor cell seeding. Student's t-test was used for the p-values. **F.** Immunofluorescence analysis of pMLKL staining of vehicle (DMSO) and NSA treated MDA-MB-231 tumors. The scale bars represent 50 μm. **G.** Quantitative estimates of the fraction of pMLKL positive cells in tumor sections from DMSO and NSA treated tumors. Error bars represent SEM, n=10. Five randomly chosen fields from two tumor sections were chosen. Student's t-test was used for p-value calculation.

Thus far our experiments suggest a clear role for all three necroptotic genes in sustaining the tumorigenicity of malignant cells. We next carried out experiments to examine whether the chemical compound necrosulfonamide (NSA) had any anti-tumor efficacy. NSA is a specific inhibitor of human MLKL. NSA covalently modifies Cys86 of human MLKL to prevent the RIPK1-RIPK3-MLKL necrosome formation and thereby blocking its interaction with downstream effectors of necroptosis [12]. NSA has been shown to be effective in preventing TNF-α induced necroptosis. However, our results in Figures 1&2 suggest that NSA might attenuate the tumorigenic abilities malignant cells. In order to examine this possibility, we evaluated the effect of NSA on soft agar clonogenic abilities of MDA-MB-231 cells. Our results indicate that, at concentrations (≤2.5μM) that did not have significant growth-suppressive effect on cell growth (Supplementary Figure S7), NSA could effectively suppress anchorage-independent MDA-MB-231 colony formation in soft agar (Figure 3A&3B). Similar results are shown in NSA treated HCT116 tumor cells (Supplementary Figure S8). In addition, NSA could also effectively reduce the basal level of MLKL phosphorylation (Figure 3C). Most interestingly, when NSA was administered *in vivo* (7 daily injections of NSA at 3.7 mg/kg/day, i.p.), it could significantly suppress xenograft tumor formation from MDA-MB-231 cells in nude mice. Both tumor growth curve (Figure 3D) and tumor weight measurements at the end of the experiments (Figure 3E) suggested potent tumor-suppressive efficacy of NSA. Figure 3F shows the immunofluorescence analysis of pMLKL staining in MDA-MB-231 tumors with or without NSA treatment. NSA significantly inhibited MLKL phosphorylation in xenograft MDA-MB-231 tumors, as expected (Figure 3G). One caveat of the above experiments is that NSA may possess off-target effects in additional to MLKL. Additional experiments evaluating NSA in *MLKL* KO xenograft are required to examine such a possibility.

### Identification of NF-κB regulated cytokine secretion as a key mechanism mediating the tumor–promoting effects of necroptotic factors

**Figure 4:**
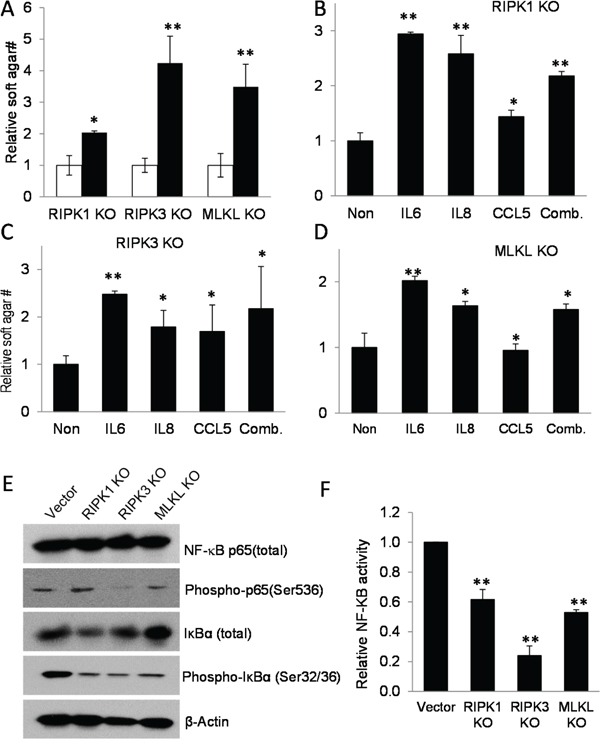
Cytokines regulated by necroptotic genes play key roles in sustaining tumor cell growth **A.** Relative soft agar growth from MDA-MB-231 cell deficient in RIPK1, RIPK3, or MLKL cells without (□) or with (■) supernatant supplements from parental MDA-MB-231 cells at 40% of culture medium in soft agar plates. *, p<0.005, **, p<0.001, Student's t-test. **B.** The effect of adding IL6 (20 ng/nl), IL8 (10 ng/ml), or CCL5 (5 ng/ml) individually or in combination(Comb.) on soft agar growth in RIPK1 knockout cells. *p<0.05, **, p<0.001, Student's t-test. **C.** The effect of adding IL6 (20 ng/nl), IL8 (10 ng/ml), or CCL5 (5 ng/ml) individually or in combination(Comb.) on soft agar growth in RIPK3 knockout cells. *p<0.05, **, p<0.001, Student's t-test. **D.** The effect of adding IL6 (20 ng/nl), IL8 (10 ng/ml), or CCL5 (5 ng/ml) individually or in combination(Comb.) on soft agar growth in MLKL knockout cells. *p<0.05, **, p<0.001, Student's t-test. **E.** Western blot analysis of key NF-κB proteins p65 and IκBα. Vectors transduced MDA-MB-231 cells and those with the necroptotic gene knockouts were analyzed for total and phosphorylated p65 and IκBα status. **F.** Significantly attenuated NF-κB reporter activities in RIPK1-, RIPK3-, or MLKL- knockout MDA-MB-231 cells compared with vector-transfected MDA-MB-231 cells. NF-κB activities were assayed by use of a lentiviral vector encoding a reporter that consists of a NF-κB responsive promoter controlling a firefly luciferase gene. **, p<0.001, Student's t-test. Error bars in C-E, and G represent SEM (n=3).

We next attempted to understand how the necroptotic genes sustain the tumorigenic abilities of the MDA-MB-231 cells. As we observed that during clonogenic and soft agar growth experiments, *RIPK3*-, *RIPK1*-, or *MLKL*- knockout cells tend to have significantly smaller colonies (Supplementary Figure S3), we hypothesized that the necroptotic genes may regulate important cytokines required for tumor cell growth. We therefore did rescue experiment by adding supernatant from the wild type MDA-MB-231 cells to the knockout cells in soft agar growth experiments. Our results indicate the supernatant from the parental cells significantly boosted soft agar colony growth of cells with the necroptotic gene knockouts (Figure 4A). Our results therefore confirmed that there are paracrine factors secreted by the parental cells that are missing in necroptotic gene knockout cells. In order to identify which factors are missing, we carried out a cytokine profiling analysis (Supplementary Figure S9) by use of a commercially available array with antibodies against over 100 known cytokines. Our results indicate there were indeed multiple cytokines that were missing in the supernatants of knockout cells.

A literature examination of the missing cytokines (IL6, IL8, LCN2, MCP1, and CCL5) indicated that most of them have indeed been implicated in tumor growth, tumor angiogenesis, or tumor invasion [46–51]. In order to confirm the cause-effect relationship between the missing cytokines and reduced tumorigenic abilities, three of the cytokines (IL6, IL8, and CCL5) were added individually and in combination to the knockout MDA-MB-231 cells in soft agar growth assays. Our results indicate that indeed, the cytokines can rescue soft agar growth of the knockout cells in soft agar (Figure 4B-4D). Our results therefore support the importance of these cytokines in promoting anchorage-independent cell growth of MDA-MB-231 cells.

How do RIPK1, RIPK3, and MLKL regulate pro-growth cytokine secretion? In order to answer that question, we decided to examine NF-κB. NF-κB is a central regulator of inflammatory cytokine secretion [52, 53]. Previous studies have shown that it is a key transcriptional regulator of IL6, IL8, and CCL5 cytokines. More importantly, both RIPK1 and RIPK3 have been implicated in NF-κB regulation [54–56]. Western blot analysis (Figure 4E) indicate the phosphorylation of IκBα was significantly reduced in the three knockout cell lines when compared with control, vector-transduced cells. Since IκBα is an inhibitor of NF-κB and its phosphorylation is correlated with activation of the NF-κB transcriptional activity, the reduction of IκBα phosphorylation is consistent with reduced NF-κB activity in the knockout cells. Our analysis (Figure 4E) also indicated that p65/RelA phosphorylation, a marker of NF-κB activation, was clearly reduced in *RIPK3* knockout cells but remain unchanged on *RIPK1* and *MLKL* knockout cells. So the overall results appear to suggest that NF-κB activities should be reduced in the knockout cells to different degrees. Indeed, when we used an artificial NK-κB reporter to determine its activities in the parental and necroptotic gene knockout cells, we found that NF-κB activities were significantly attenuated in the knockout cells (Figure 4F), thereby supporting impaired NF-κB activities as a mechanism through which missing necroptotic factors cause reduced inflammatory cytokine secretion in MDA-MB-231 cells.

### Relationship between tumor pMLKL levels and patient survival

**Figure 5:**
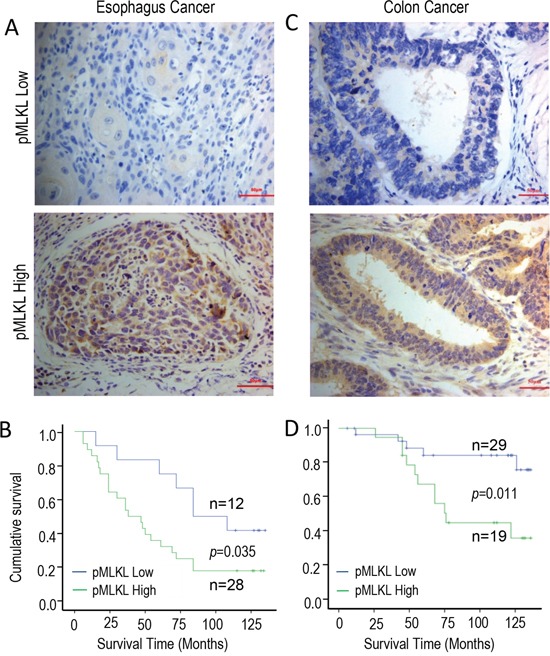
Kaplan-Meier survival of analysis of the relationship between cumulative patient survival and phosphorylated MLKL levels (derived from IHC analysis) in pre-treatment human tumor samples **A.** Examples of low (top panel) and high (lower panel) pMLKL staining in human esophageal cancer samples. The scale bar represents 50 μm. **B.** Kaplan-Meier analysis of the relationship between cumulative survival and pMLKL staining levels in human esophageal cancers (n=40, p=0.035, log-rank test). **C.** Examples of low (top panel) and high (lower panel) pMLKL staining in human colon cancer samples. The scale bar represents 50μm. **D.** Kaplan-Meier analysis of the relationship between cumulative survival and pMLKL staining levels in human colon cancers(n=48, p=0.011, log-rank test). Please see Methods section and Table S2 for more information on the staining methods and patient information.

We next examined the relevance of our discovery of the tumor growth-promoting properties of the necroptotic genes in human cancer patients. In order to do this, we used immunohistochemistry to determine the levels of phosphorylated MLKL (pMLKL) in pre-treatment biopsy samples from a cohort of human esophagus cancer patients and a cohort of human colon cancer patients (Figure 5, Supplementary Table S2). Our results indicate that tumor samples from both cohorts exhibit low (Figure 5A & 5C, top panels) and high (Figure 5A & 5C, lower panels,) levels of staining for phosphorylated MLKL. Most interestingly, higher levels of pMLKL staining in both the esophageal (Figure 5B) and colon (Figure 5D) cancer samples correlated with significantly lower survival levels among the patients. Those results are consistent with our observation that activated necroptotic factors play a key role in sustaining human malignancies.

## DISCUSSION

In our study, we evaluated the effects of the necroptotic genes *RIPK1, RIPK3*, and *MLKL* in tumor growth and tumor resistance to therapy. Despite suggestions in previous publications that expression and activation of these genes may promote tumor cell death and therefore beneficial for tumor therapy, we provide strong evidence that the necroptotic genes in fact play critical roles in maintaining the tumorigenic abilities of cancer cells and their resistance to radiotherapy. Our experiments indicate that the roles of necroptotic genes in promoting inflammatory cytokine production and thereby sustaining tumor growth appear to outweigh that of promoting necroptotic cell death. Importantly, our data also show that higher phosphorylation levels of MLKL, a key necroptotic factor, correlated with shorter human patient survival.

Even though our results are surprising, they are consistent with recent results from studies conducted on two other forms of cell death, apoptosis [57] and autophagy [58] that demonstrated cell death pathways may actually promote tumor growth. They show that the simple assumption of all death-promoting process/factors being beneficial to cancer treatment needs a much more nuanced examination. Multiple studies have now indicated that pro-apoptotic factors and autophagic factors may in many instances be pro-carcinogenic or pro-tumor survival. For example, it was found that apoptotic caspase 3 activation in dying tumor cells can activate paracrine pro-survival during radiotherapy [57]. Importantly, it was also demonstrated that higher levels of activated caspase 3 correlated with poor patient survival in breast cancer and head and neck cancer patients [57]. Therefore, the overall effect of caspase 3 activation appears to be negative for cancer treatment in those two instances. In other published studies, it was shown that CD95(Fas), a well-established pro-apoptotic factor, can promote carcinogenesis in mice [59]. Similarly, studies on factors involved in autophagy indicate that in many instances it protects rather than kills cancer cells in radiation or chemotherapy [60, 61]. Our study is also consistent with a recent study indicating RIPK1 being a driver for melanoma growth [62].

How can we understand the paradoxical observations that factors in three forms of cell death, apoptosis, necrosis, or autophagy, all appeared to play pro-tumor survival roles at least under some circumstances? The main confusion appears to lie in the fact that in all three instances the common perception is that activation of death-promoting factor equals to cell death and elimination of the cancer cell. However, two observations from recent studies might explain the discrepancy between what's expected and what's observed. First, activation of death-promoting factors may not always leads to cell death [63, 64]. Examples of this include caspase 3 or autophagy factors. Cells that survive the activation in some instances become transformed [63, 64] or are better equipped to deal with the external stress. Second, even those cells which are destined to die can secret growth-promoting signals, such as caspase 3-mediated signaling published earlier [57] or the necroptotic factor-regulated signaling described in the present study. For these reasons, the overall effect of activation of cell death signaling on tumor growth is often contrary to expectations from the established paradigm.

Our study has mainly focused on NF-κB as a key downstream effector of the necroptotic factors. This was mainly based on our cytokine array profiling, which suggest the involvement of NF-κB. However, it possible that other factors may be involved. One such factor is the NLRP3 inflammasome [65, 66], which is involved in regulation of IL-1β. The latter has been shown to be a potent stimulator of tumor angiogenesis and tumor growth [67–69]. It is also possible that additional, yet unidentified pathways may be involved. To identify such pathways, unbiased genomic or proteomic profiling of the wild type and knockout cells may be necessary.

One interesting implication for the present study is the potential to use anti-necroptotic agents to enhance cancer therapy. Our data with a previously published inhibitor of MLKL (NSA) certainly illustrated the great potential of this approach. Considering the fact that necroptotic gene knockout also appears to sensitize breast cancer cells to radiotherapy, it is also reasonable to expect that agents that inhibit necroptotic factors may also be combined with radiotherapy to enhance the efficacy of the latter. The fact that human tumor samples that stained positive for pMLKL are correlated with worse patient survival suggests that our discovery also has human patient relevance. Although further animal testing and chemical inhibitors with better pharmacokinetic properties might be necessary to justify a clinical trial, the prospect of using inhibitors of necroptosis in human cancer treatment certainly looks promising.

## MATERIALS AND METHODS

### Cell culture

MDA-MB231 is a triple-negative breast cancer cell line. 4T1 is spontaneous mouse breast cancer line. HCT116 is a colon cancer cell line. All three were obtained from the Tissue Culture Shared Resource of the Duke Cancer Institute. The cells were maintained in DMEM supplemented with fetal bovine serum and penicillin-streptomycin.

### CRISPR-mediated knockout

Tumor cells deficient in various knockout genes were made by use of the CRISPR/Cas9 technology [40, 41]. Target single guided RNA (sgRNA) sequences were generated with the use of a free online CRISPR design tool (crispr.mit.edu). The sgRNA sequences are shown in Supplementary Table S1. Annealed double stranded sgRNA oligos were ligated into the lentiCRISPR vector (deposited by Dr. Feng Zhang to Addgene, Cambridge, MA) at BsmBl site, which co-express cas9 and sgRNA in the same vector. The constructed CRISPR vectors were prepared, packaged according the manufacturer's protocol. The human breast cancer cell, MDA-MB-231 or mouse breast cancer cell, 4T1 were infected with CRISPR lentivirus vector and cultured in DMEM medium supplemented with 10% FBS, and 1 μg/ml purimycin and incubated at 37°C with 5% CO_2_ in a humidified cell-culture incubator for 14 days. Then, the infected cells were plated to 96-well plates with 1 cell per well. The clonal cell populations became visible, they were transferred and expanded for western blot detection. Those clones that showed disrupted protein expression were then subjected sequencing verification of gene disruption. Supplementary Table S1 shows the primers used to amplify the target gene sequences surrounding the target gene site. Once amplified, the PCR products were verified by Sanger sequencing. The sequence primers used were the same as those used as forward primers in the PCR reactions.

### Western blot analysis

Cultured cells were lysed by use of the standard RIPA lysis buffer. Total cellular proteins were then separated by SDS-PAGE gel electrophoresis. The primary antibodies used were mouse anti-RIPK1 (R&D), rabbit anti-RIPK3 (Abcam), rabbit anti-MLKL (Millipore), and rabbit anti-phospho-MLKL (Millipore). The primary NF-kB antibodies, rabbit anti-NF-kB p65, rabbit anti--phospho-NF-kB p65, mouse anti-IkBα, rabbit anti--phospho-IkBα, were obtained from Cell signaling Technology. The secondary antibodies used were goat anti-mouse or rabbit IgG (Jackson Immuno Research).

### Assays for cellular growth, clonogenic survival, and soft agar growth

For cell growth assay, the cells were plated at a density of 1,000 cells per well in 96-well plates and stained daily by use of the MTT (3-(4,5-Dimethylthiazol-2-yl)-2,5-Diphenyltetrazolium Bromide) reagent (ATCC). Cellular densities were then quantified by use of a Biotek Synergy H1 plate reader. Triplicate wells were plated for each cell type.

For clonogenic survival assay, an established protocol was used [42]. Briefly, the cells were irradiated with different doses of x-rays and then plated in triplicate at different numbers according to different radiation doses so that there were 50-200 colonies form eventually in 10-cm dishes. After colonies are clearly visible after 10-14 days, cells were fixed and stained with 0.5% crystal violet, rinsed and dried. Colony numbers are then enumerated and the surviving fractions were calculated according to the number of initially plated cell numbers and the plating efficiency in un-irradiated control cells.

For soft agar assay, an established protocol was used [45]. Cells were seeded at a density of 250 cells per well in 6-well plates in triplicate. To evaluate the soft agar growth effect of necrosulfonamide (NSA, TOCRIS), the cells were cultured with different concentrations of NSA. To rescue the tumorigenic abilities of knockout MDA-MB-231 cells, the supernatant from wild type tumor cells or several of the human recombination cytokines (20 ng/ml IL6, R&D;10 ng/ml IL8, R&D; and 5 ng/ml CCL5, R&D) were added individually and in combination to knockout MDA-MB-231 cells. After development of colonies at 21 days, the cells were fixed and stained with 0.005% crystal violet. Images of each well were taken. The number of colonies per well were quantified by use of Image J (NIH).

### Radiation exposure

Cells were irradiated *in vitro* with an X-RAD320 biological irradiator (Precision X-Ray) with different doses at 320 kVp, 12.5 mA X-rays. Radiation dose rates were measured and calculated by personnel from the Radiation Safety Division of Duke University.

### Cytokine array

To profile cytokines present in the supernatant of parental and knockout MDA-MB-231, the cells were plated at a density of 100,000 cells per well in 6-well plate and allowed to adhere overnight. Medium was then changed to 1 ml with 1% FBS. The supernatant was collected at 48h. The cytokines were then profiled according to manufacturer's instruction that comes with Human Cytokine Assay Kit (R&D).

### Real-time PCR

To quantify the amount of cytokine mRNA in parental as well as knockout MDA-MB-231 cells, total RNA was isolated form the cells by use of the RNeasy mini kit (QIAGEN). Reverse transcription of mRNA was then carried out with the superscript III cDNA synthesis kit (Invitrogen). Quantitative Real-time PCR was performed by use of the SsoAdvanced universal SYBR Green supermix (Bio-Rad) for the mRNA of *IL6, IL8, LCN2, MCP1*, and *CCL5*. GAPDH mRNA level was used as an internal control to normalize the concentration of cDNA in different samples. The primers for amplifying the cytokine genes were shown in Supplementary Table S1. Triplicate samples were measured for each cell line.

### NF-κB reporter assay

To evaluate the activities of the transcriptional factor NF-κB in parental and knockout MDA-MB-231 cells, we obtained a lentivirus-based pGreenFire-NF-κB reporter from System Biosciences. The lentiviral vector expresses a destabilized copGFP gene and a firefly luciferase gene under control of four NF-κB response elements (GGGACTTTCC) and a minimal CMV promoter. It also has a neomycin resistance gene for selection of cells that have integrated the lentiviral vector.

### Tumor growth delay experiments in mice

All animal experiments were approved by the Duke University Institutional Animal Care and Use Committee (IACUC). Human patient tumor sample analyses were approved by the West China Second University Hospital Ethics Committee for Medical Research.

For MDA-MB-231 xenograft tumor growth, we obtained 6-week old female nude mice from Taconic and maintained them at the Duke University Vivarium. For tumor formation, about 2 × 10^5^ vector transfected or knockout (RIPK1-, RIPK3-, or MLKL-KO) MDA-MB-231 cells were injected into the hind limb of the nude mice subcutaneously. The mice were then monitored for tumor growth on a daily basis. Tumor sizes were measured by use of a caliper.

For evaluating the effect of RIPK3 knockout on 4T1 tumors, about 2 × 10^5^ vector-transduced or RIPK3 knockout 4T1 cells were injected into the hind limb of 6-week old female Balb/C mice (Taconic) subcutaneously. Tumor sizes were then measured every 2-3 days.

To evaluate the anti-tumor effect of NSA, 2 × 10^5^ MDA-MB-231 cells were injected subcutaneously into 6-week old female nude mice. When tumors reach 2-3 mm in diameter, they were injected either with vehicle (10% DMSO in PBS) or NSA (Tocris) at the dosage of 3.7 mg/kg i.p. on a daily basis for 7 days. The sizes of the tumors were measured twice a week with a caliper.

### Immunofluorescence and immmuohistochemistry analysis

Upon termination of tumor growth experiments, 4T1 or MDA-MB-231 tumors were excised and fixed in 10% formalin and paraffin-embedded. Embedded tumors were then sectioned and subjected to H&E staining and immunofluorescence staining. To visualize phosphorylated MLKL, a recently established marker for necroptosis, we used a rabbit anti-phopho-MLKL (Abcam) primary antibodies. Secondary antibodies used were goat goat anti-rabbit (alexa fluor 488) at 1:500 dilution (Invitrogen). Images were acquired with a ZEISS fluorescence microscope (Zeiss). Quantification of the fraction of stained cells was performed by a technician blinded to genotype and treatment with the aid of the Image J (NIH) software.

### Analysis of pMLKL levels in human patient-derived tumor samples

Two cohorts of human cancer patient samples from West China First University Hospital, Sichuan University, were analyzed. See Table S2 for patient characteristics. To analyze the levels of pMLKL in human cancer patients, paraffin-embedded, pretreatment tumor sections were IHC-stained with an anti- phopho -MLKL antibody (Abcam). The stained sections were then scored as follows: 0 (0-9% tumor cells stained), 1 (10%-30% positive tumor cells), 2 (31%-50% positive tumor cells), 3 (51%-80% positive tumor cells), 4 (81%-100% positive tumor cells). Three photos (×400) were taken randomly for each specimen and the final immunoreactivity score (IS) for each specimen was the average value of the three photos. Using the value 2 of IS as a cut-off value, pMLKL expression was divided into pMLKL- high (IS ≥ 2) and pMLKL-low (IS < 2) groups.

### Statistics

Statistical analyses of experimental results in this study were conducted by use of a variety of statistical tests that included 2-tailed Student's t test, one-way ANOVA, or two-way ANOVA. For Kaplan-Meier analysis, the log-rank test was used to compare the differences in cumulative survival between patient groups.

## SUPPLEMENTARY FIGURES AND TABLES


