# Supplementary material for: Key roles of necroptotic factors in promoting tumor growth
Source: Oncotarget. 2016 Mar 5;7(16):22219–33. doi: 10.18632/oncotarget.7924 (PMC5008357; doi:10.18632/oncotarget.7924)
Supplement: Supplementary file 1 [file oncotarget-07-22219-s001.pdf]

SUPPLEMENTARY FIGURES AND TABLES

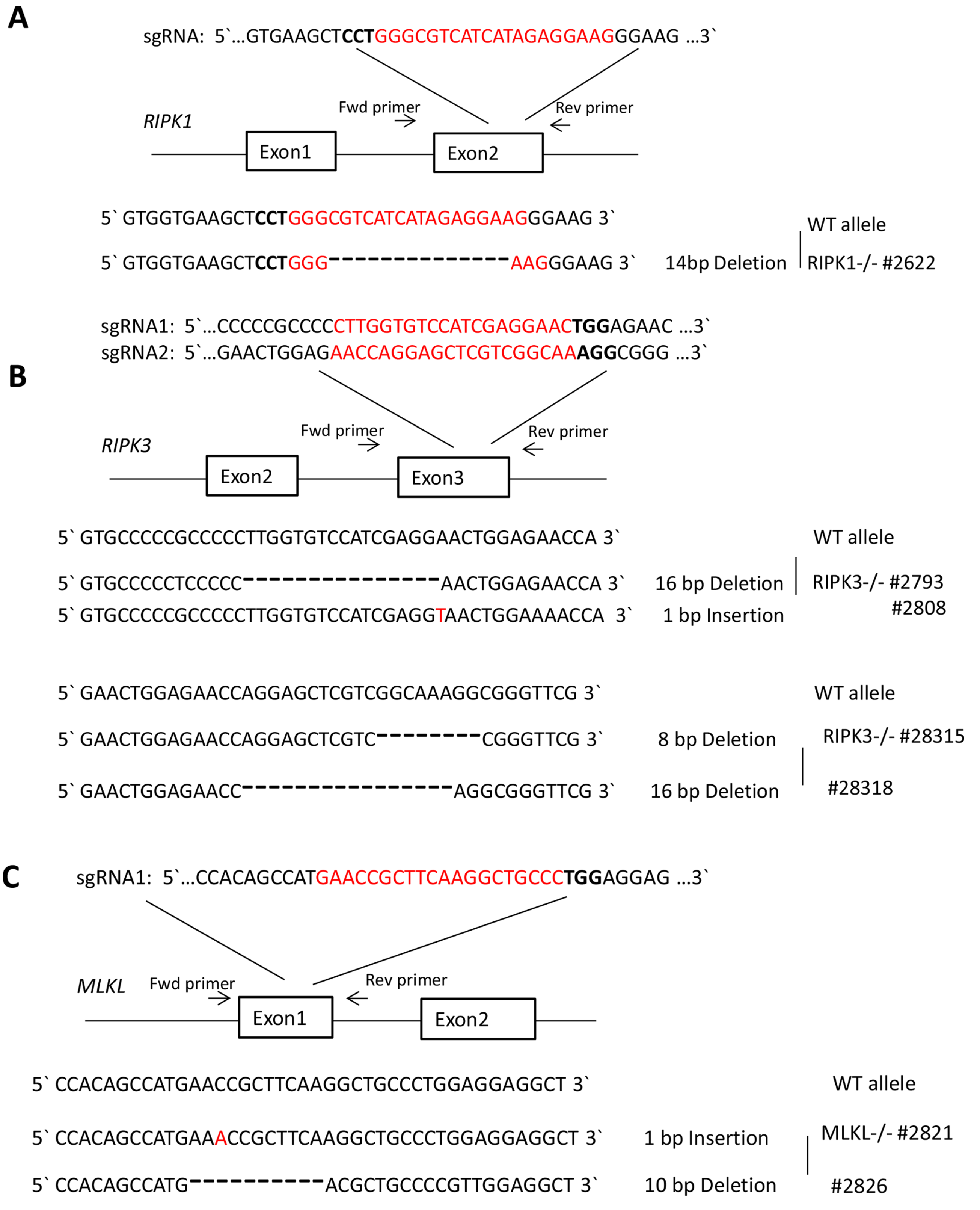

Supplementary Figure S1: Detailed sgRNA and gene mutation information for MDA-MB-231 cells with *RIPK1*. A. *RIPK3* **B.** and *MLKL* knockouts **C.** The examples shown are all with homozygous mutations that caused the same frameshifts in both copies of the target genes. Each number represents a single clone.

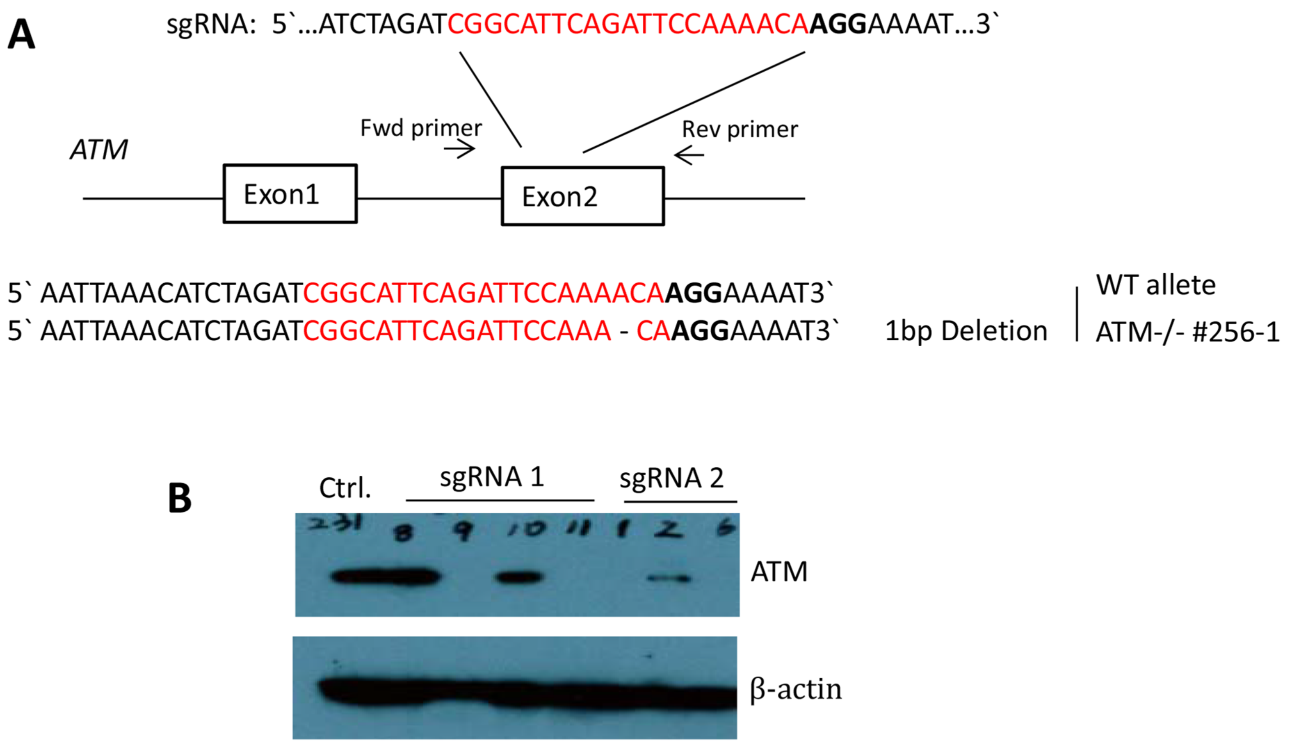

Supplementary Figure S2: A. sgRNA target information and gene mutation information for a MDA-MB231 clone with ATM gene knockout. **B.** Western blot analysis of ATM protein expression levels in individual MDA-MB-231 clones with transduced with sgRNA targeted to ATM.

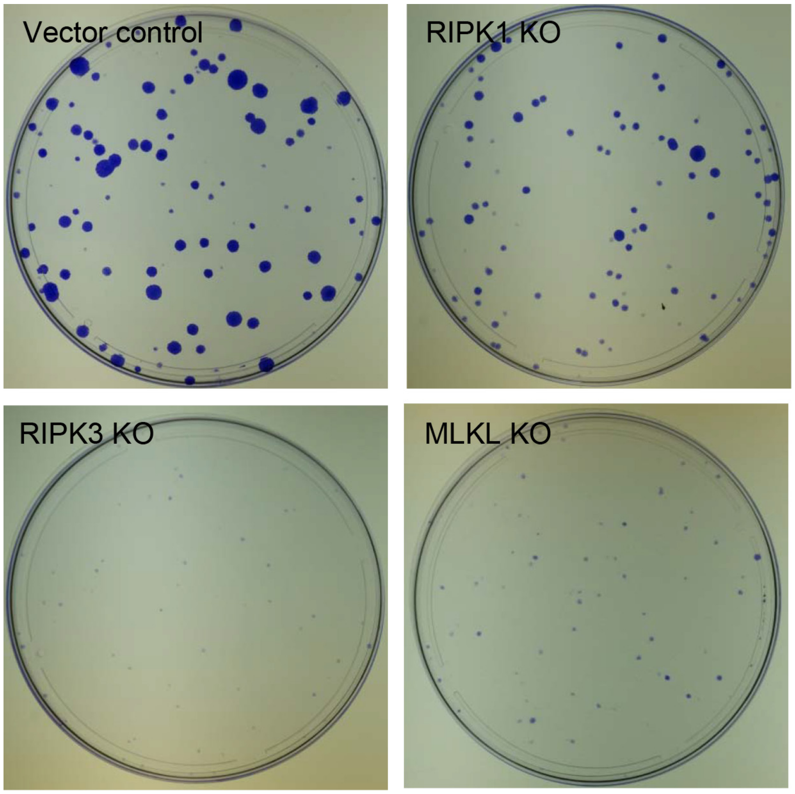

Supplementary Figure S3: Colony sizes from MDA-MB-231 cells with vector-transduced, or those with RIPK1-, RIPK3-, and MLKL- knockouts. About 200 cells were plated in 10-cm Petri dishes. The photographs were taken at 2 weeks post seeding. Notice the different colony sizes among the control and knockout cells.

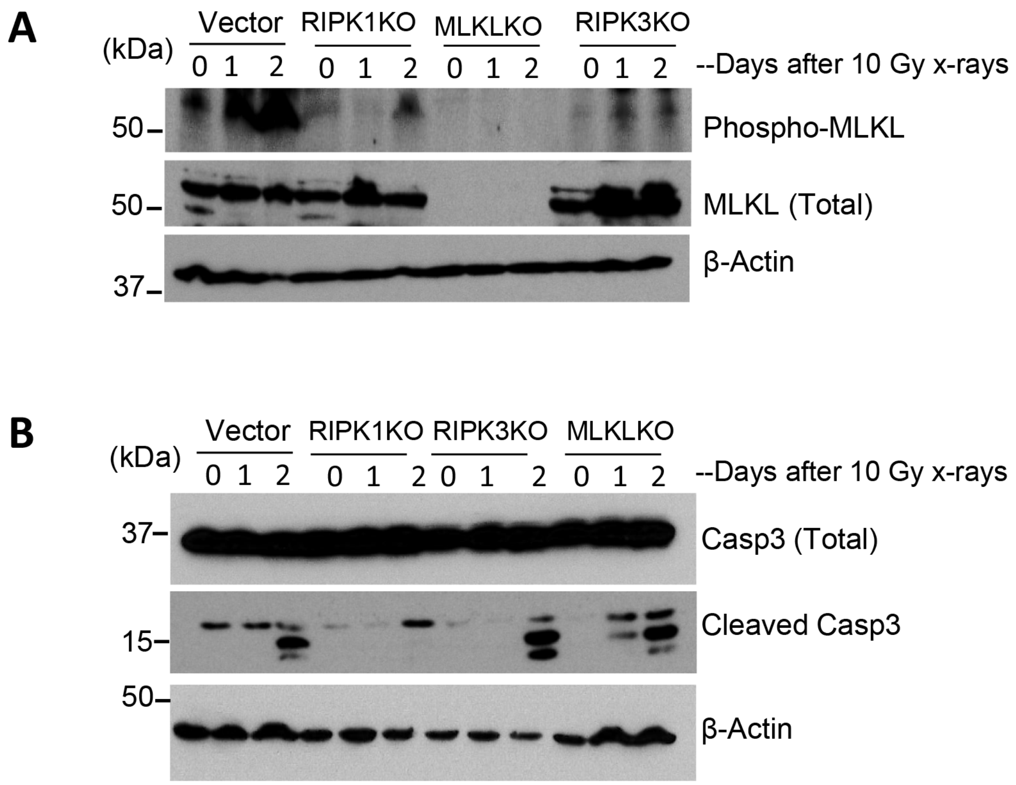

Supplementary Figure S4: Analysis of various cell death markers in MDA-MB-231 cells with necroptotic gene knockout. A. Western blot analysis of phosphorylated MLKL (top panel), total MLKL (middle panel), and β-actin (lower panel) in cells that has been exposed to 10 Gy of x-rays. Zero time indicated sham-irradiated cells. **B.** Western blot analysis of total, cleaved caspase 3, and β-actin in vector-transduced control MDA-MB-231 and knockout cells at 24 and 48 hrs after exposure to 10 Gy of x-rays. Zero time indicate sham-irradiated cells.

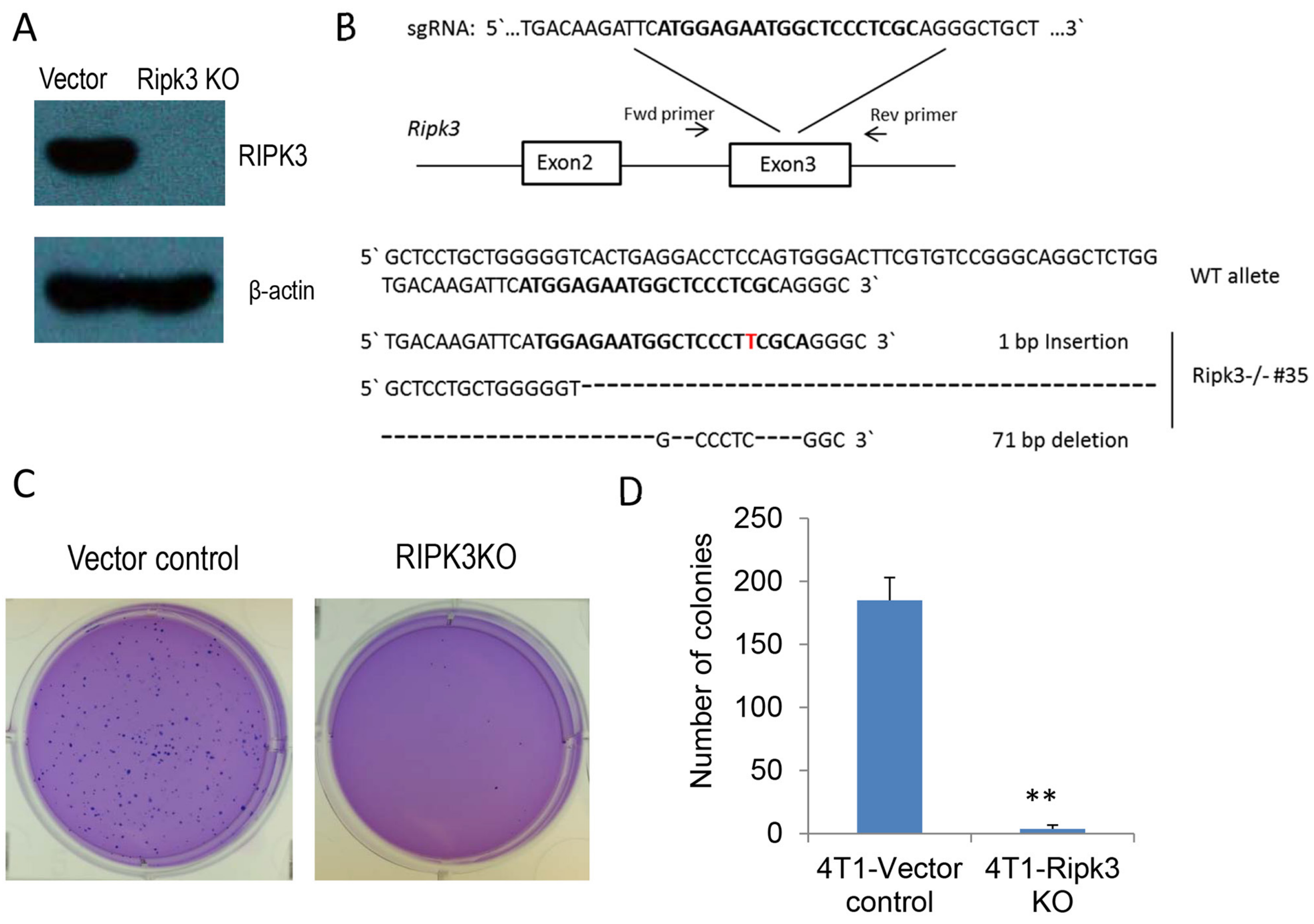

Supplementary Figure S5: A. Western blot analysis of and *RipK3* knockout 4T1 cells. **B.** Sequencing verification of a knockout 4T1 cell line with heterozygous Ripk3 gene knockout mutations. **C.** Representative soft agar colony growth from 4T1 cells transduced with vector control and those that showed RIPK3 deficiency. Five hundred cells were plated into each soft agar plate. **D.** Quantitative estimate of soft agar colonies from control 4T1 and RIPK3-KO 4T1 cells. The error bars represent the standard error of the mean (SEM, n=3, **p<0.001, Student's t-test).

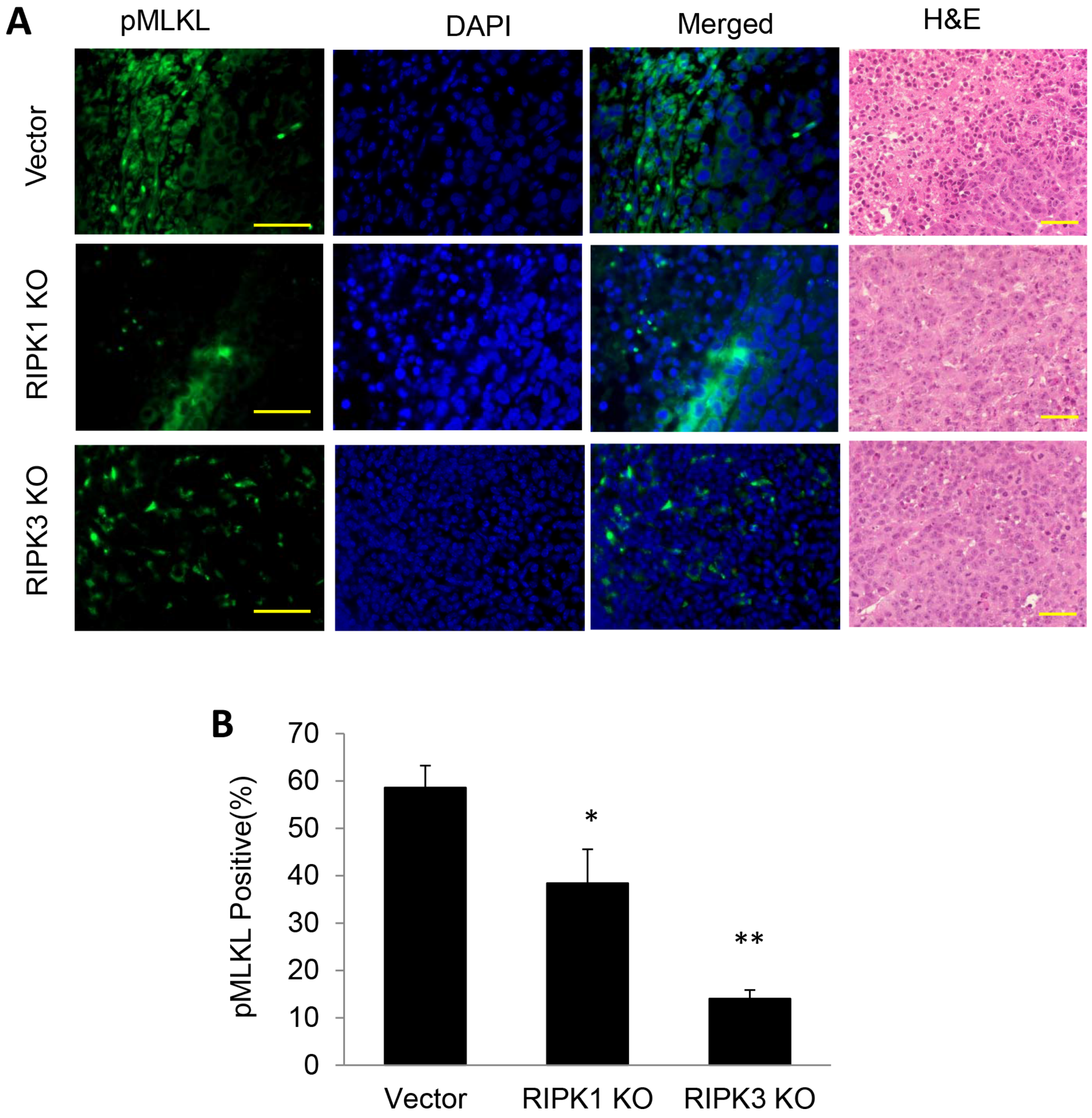

Supplementary Figure S6: Immunofluorescence staining of phosphorylated MLKL (pMLKL) in MDA-MB-231 xenograft tumors. A. Representative immunofluorescence staining of pMLKL in tumors derived from MDA-MB-231 cells transfected with vector control, RIPK1-KO, and RIPK3-KO cells (left 3 columns). The right most column shows H&E staining of adjacent areas of the immunofluorescence staining The scale bars represent 50 µm. **B.** Quantitative estimate of the percentage of tumor cells that stain positive for pMLKL. Error bars represent SEM. Each data point is derived from the average of counting 10 randomly chosen areas from two tumor sections (five areas from each section were counted). *, $p<0.05$; **, $p<0.001$, Student's test.

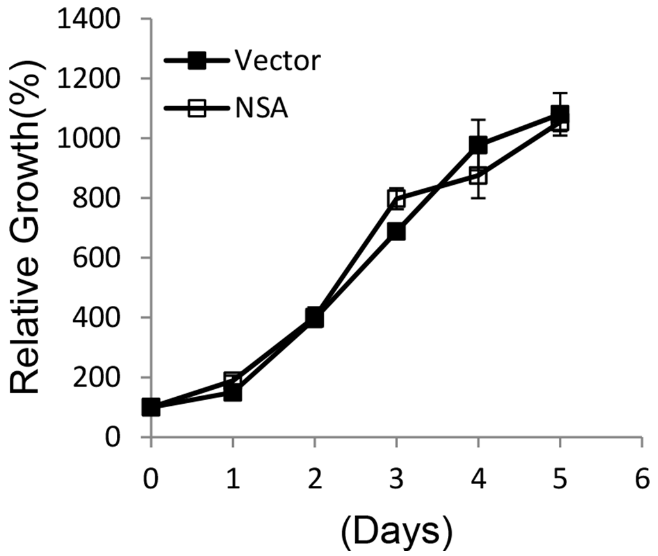

Supplementary Figure S7: Cell growth curve from MDA-MB-231 cells exposed to vehicle (DMSO) and NSA (at the concentration of 2.5 µM). Error bars represent standard error of the mean. No significant difference was observed between the two groups.

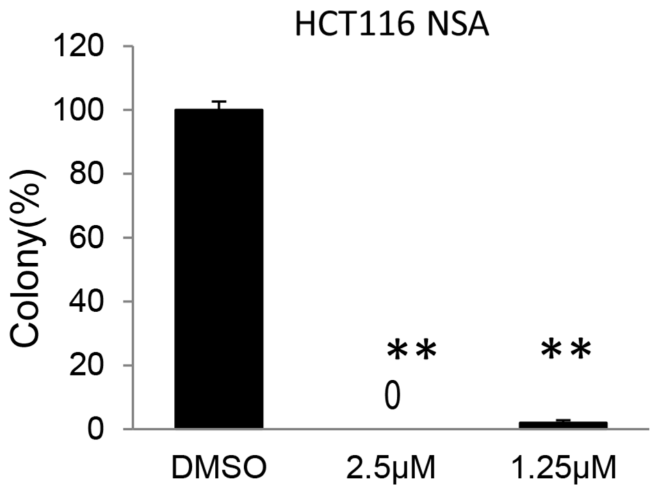

Supplementary Figure S8: Relative fraction of soft agar colony growth of NSA treated HCT116 cells. Error bars represent SEM (n=3, **, p<0.001, Student's t-test).

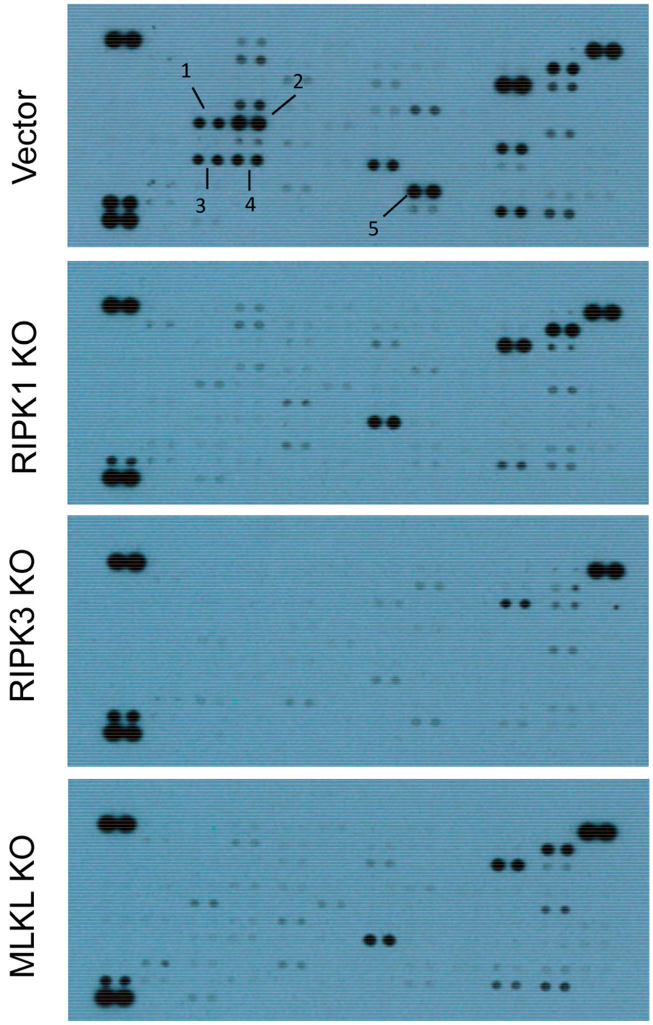

Supplementary Figure S9: Cytokine array analysis of the supernatants from vector-transduced MDA-MB-231 cells and cells with gene knockouts in RIPK1, RIPK3, and MLKL. The dots labeled with numbers in the top panel represent those that appeared to be significantly attenuated in the knockout cells. The identities of cytokines are: 1, IL6; 2, IL8; 3,LCN2; 4,MCP1; 5,CCL5.

Supplementary Table S1: Oligos used in the study

sgRNA Oligos	Oligo (5'-3')
hRIPK1sgRNA 1F	CACCGcggctttcagcacgtgcatc
hRIPK1sgRNA 1R	AAACgatgcacgtgctgaaagccgC
hRIPK1sgRNA 2F	CACCGcttcctctatgatgacgccc
hRIPK1sgRNA 2R	AAACgggcgtcatcatagaggaagC
hRIPK3sgRNA 1F	CACCGctcgtcggcaaaggcgggtt
hRIPK3sgRNA 1R	AAACaacccgcctttgccgacgagC
hRIPK3sgRNA 2F	CACCGcagtgttccgggcgcaacat
hRIPK3sgRNA 2R	AAACatgttgcgcccggaacactgC
hMLKLsgRNA 1F	CACCGaagaaacagtgccggcgcct
hMLKLsgRNA 1R	AAACaggcgccggcactgtttcttC
hMLKLsgRNA 2F	CACCGcacaccgtttgtggatgacc
hMLKLsgRNA 2R	AAACggtcatccacaaacggtgtgC
mRIPK3sgRNA F	CACCGgtgggacttcgtgtccgggc
mRIPK3sgRNA R	AAACgcccggacacgaagtcccacC
Primers for genomic PCR	
RIPK1 sg1,2F	AGGAAGTGAGAAACCGCTCTG
RIPK1 sg1,2R	AGGGCAGTAAAAGGCAAGGAA
RIPK3 sg1,2F	GCAGCCTCCGACATTCACA
RIPK3 sg1,2R	GATAACCCCTTCTAGGCGCA
MLKL sg1,2F	CAGCTCTTCCCAGCTACAACT
MLKL sg1,2R	ATTCAACTGCACTCCCACTCC
Primers for real time PCR	
IL6 F	GAACTCCTTCTCCACAAGCGCCTT
IL6 R	CAAAAGACCAGTGATGATTTTCACCAGG
IL8 F	TCTGCAGCTCTGTGTGAAGG
IL8 R	ACTTCTCCACAACCCTCTGC
LCN2 F	AGACAAAGACCCGCAAAAG
LCN2 R	TGGCAACCTGGAACAAAAG
MCP1 F	CCCCAGTCACCTGCTGTTAT
MCP1 R	TGGAATCCTGAACCCACTTC
CCL5 F	CGTGCCCACATCAAGGAG
CCL5 R	GGACAAGAGCAAGCAGAAAC
GAPDH F	GTGGACCTGACCTGCCGTCT
GAPDH R	GGAGGAGTGGGTGTCGCTGT

Supplementary Table S2: Clinical characteristics of esophagus and colon cancer patients

	Esophagus Cancer			Colon Cancer		
	pMLKL		*p*	pMLKL		*p*
	Low	High		Low	High	
Age			0.41			0.507
≤50y	3	4		8	6	
>50y	9	24		21	13	
Sex			1			0.033
male	10	24		14	15	
female	2	4		15	4	
Stage			0.24			0.060
I+II	8	13		24	11	
III+IV	4	15		5	8	
Lymph node			0.185			0.013
positive	5	18		4	9	
negative	7	10		25	10	
Grade			0.038			0.005
1+2	9	11		27	11	
3	3	17		2	8	
